# Pembrolizumab-Induced Colitis and Diarrhea in the Treatment of Sporadic Colorectal Cancer: A Case Report

**DOI:** 10.7759/cureus.52636

**Published:** 2024-01-20

**Authors:** Rejath Jose, Jasveen Kaur, Theophany Blanco, Samantha Ehrlich, Michael Marcelin

**Affiliations:** 1 Internal Medicine, New York Institute of Technology (NYIT) College of Osteopathic Medicine, New York, USA; 2 Internal Medicine, Maimonides Medical Center, New York, USA; 3 Gastroenterology, Maimonides Medical Center, New York, USA

**Keywords:** colorectal cancer, immune related adverse effects, pembrolizumab induced colitis, colitis, immune checkpoint inhibitor

## Abstract

Pembrolizumab is a programmed cell death receptor-1 (PD-1) blocking immune checkpoint inhibitor (ICI) that is a mainstay of cancer treatment. Pembrolizumab has a lower incidence of colitis and diarrhea compared to other ICIs. The current study presents the case of a 30-year-old female patient on pembrolizumab with stage IV colon cancer who presented with diarrhea (50 times a day) and symptoms of colitis. A computed tomography scan of the abdomen and pelvis suggested proctitis. Stool studies were negative for enteric pathogens, but stool white blood cell (WBC) was positive, and calprotectin was >10,000 ug/g. A colonoscopy showed pancolitis with small internal hemorrhoids. Histopathology showed cryptitis and crypt abscesses with mild focal architectural distortion, mucosal erosion/ulcer, and focal crypt atrophy from the cecum to the rectum. All ICIs were discontinued, and the patient was initially managed with IV fluids. The patient was subsequently started on methylprednisolone and loperamide after colonoscopy. The number of bowel movements decreased to six per day after the above management. The patient was then switched to oral prednisone and discharged with outpatient follow-up. This case reveals the importance of assessing immune-related adverse effects (irAEs) even though incidence rates associated with a specific ICI might be low.

## Introduction

Immune checkpoint inhibitors (ICIs) have become an integral part of cancer treatment regimens for many immune-sensitive tumors. The US Food and Drug Administration (FDA) has currently approved three different categories of ICIs for cancer treatment: programmed death protein-1 (PD-1) inhibitors, programmed death-ligand-1 (PDL-1) inhibitors, and cytotoxic T-lymphocyte-associated protein 4 (CTLA-4) inhibitor [1]. ICIs block the receptor-ligand interaction between different molecules that function in T-cell inhibition, allowing for more T-cell activation and subsequently destroying cancer cells [2]. The hereditary nature of colon cancer incidence allows for the use of cancer biomarkers such as high/mismatch repair deficient (MSI-H/dMMR) to assess treatment options and prognostic decision-making [3]. Pembrolizumab is FDA-approved for single-agent first-line treatment for patients with MSI-H/dMMR colorectal cancer (CRC) [4,5].

The pro-inflammatory effects of T-cell overactivation lead to several ICI-induced adverse effects that target nearly every organ system [1]. These immune-related adverse effects (irAEs) include rash, diarrhea, colitis, hepatitis, hypophysitis, and hypothyroidism [2]. Out of all the ICIs, pembrolizumab has the lowest incidence of colitis and diarrhea. Colitis secondary to pembrolizumab is only seen in approximately 1.3%-2.9% of patients, and diarrhea is seen in 2.9%-11.5% of patients [6]. There have been no previous studies detailing pembrolizumab-induced colitis and severe diarrhea in a patient with sporadic colorectal cancer along with a very elevated calprotectin. This case is particularly novel since the incidence of pembrolizumab-induced colitis, and diarrhea is lower compared to other ICIs.

## Case presentation

A 30-year-old female with a past medical history of asthma, gastroesophageal reflux disease (GERD), and stage III adenocarcinoma of the colon status post sigmoidectomy one year ago presented to the emergency department with profuse diarrhea for the past two weeks. The patient had ~50 small-volume bowel movements per day on arrival. The stool was sometimes blood-tinged and mucoid with tenesmus. Diarrhea was associated with tenderness in the right upper quadrant of the abdomen. The patient was diagnosed with adenocarcinoma one year ago and had a sigmoid resection a few months after diagnosis. Nine months after sigmoidectomy, a CT scan revealed liver metastasis, and the patient subsequently completed eight cycles of capecitabine and oxaliplatin (CAPOX), which was six months before presentation. She then completed four cycles of palliative irinotecan and capecitabine with pembrolizumab one month before the presentation. The patient had no fever, chills, chest pain, or shortness of breath. Family history was unremarkable for any colon cancer in a three-generation pedigree. Social history was not significant for smoking or alcohol use. Physical exam showed no scleral icterus with slight right upper quadrant tenderness on palpation and diffuse abdominal tenderness. Heart and lung exams were unremarkable. Vitals throughout her hospitalization/admission were within normal limits.

CT scan of the abdomen and pelvis showed a new rectal wall thickening with surrounding fat stranding, suggesting proctitis. Abdominal ultrasound was unremarkable aside from multiple hepatic metastatic lesions. Once admitted, the patient remained afebrile with minimal abdominal pain. Her blood cultures were negative. While stool studies were negative for enteric pathogens, *Clostridium difficile*, or stool ova and parasites, the stool white blood cell (WBC) count was positive, and stool calprotectin was elevated at 10,572 ug/g. An initial investigation was unremarkable for any infectious process, but a positive stool WBC indicated a possible inflammatory etiology.

**Figure 1:**
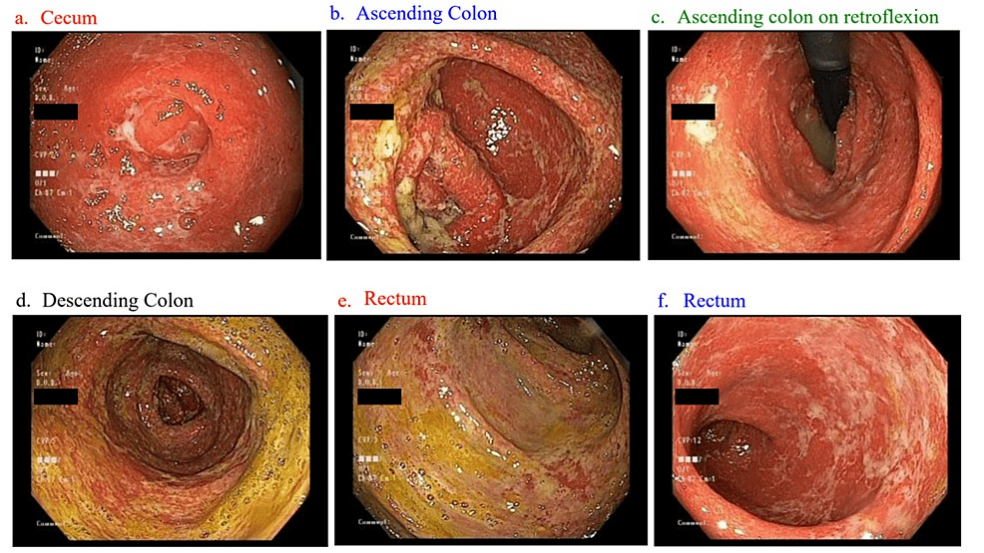
Endoscopic images showing pancolitis

A colonoscopy on hospital day 3 showed pancolitis and small internal hemorrhoids (Figure 1). Histopathology showed cryptitis and crypt abscesses with mild focal architectural distortion, mucosal erosion/ulcer, and focal crypt atrophy from the cecum to the rectum. The differential diagnosis included infectious, drug toxicity, or inflammatory bowel disease (IBD).

**Figure 2:**
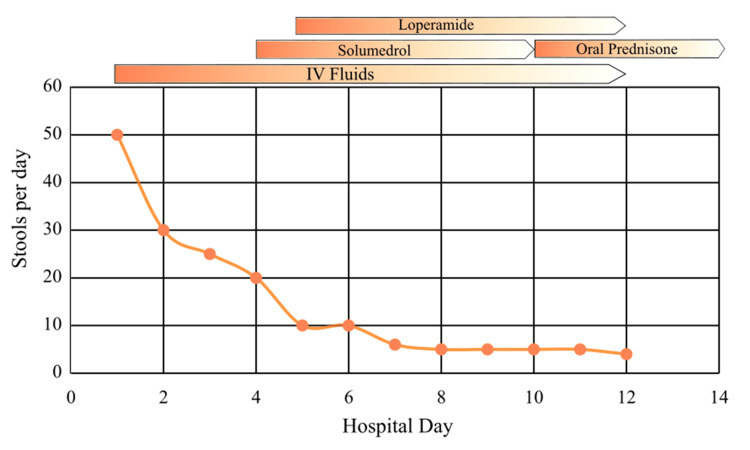
Number of stools per day in relation to interventions

In the emergency department, the patient was managed with IV fluids. On admission, all ICIs and chemotherapeutic agents were discontinued. By hospital day 3, the patient's bowel movements had decreased to 20-25 per day without any intervention. Following endoscopic evaluation on day 3, she was started on solumedrol on hospital day 4, followed by loperamide on hospital day 5. Three days after solumedrol administration (hospital day 7), the patient's bowel movements decreased to six per day. On hospital day 8, the patient was started on oral prednisone, and the number of bowel movements further decreased to five per day. Oral prednisone was continued for a few more days until the number of bowel movements was <4 (hospital day 12). Oral prednisone was continued on discharge, and the patient was scheduled to follow up with an outpatient oncologist for steroid tapering. Figure 2 shows the number of stools per day in relation to the interventions for this patient.

## Discussion

The pro-inflammatory response due to ICIs can lead to several inflammatory adverse events, especially colitis and diarrhea, which can mimic IBD or an enteric infection [7]. Colitis is diagnosed with colonoscopy findings as well as stool inflammatory markers such as stool WBC and fecal calprotectin. Diarrhea severity is often classified by the number of bowel movements per day. Clinical evidence of colitis in a patient taking any ICI should prompt the provider to stop all ICIs, work the patient for colitis, and treat accordingly. More than 80% of patients with ICI-induced colitis have endoscopic inflammatory findings like exudates, loss of vascular pattern, granular or edematous mucosa, patch or diffuse erythema, aphtha, and ulcerations [8]. Pathology findings can also be useful; however, they vary between ICIs and are often non-specific [8]. According to the National Cancer Institute Common Terminology Criteria for Adverse Events, the severity of colitis and diarrhea secondary to ICIs are graded on a scale from 1 to 5. Grade 1 colitis is asymptomatic, and grade 1 diarrhea is an increase in stools but <4 stools per day over baseline. Grade 2 colitis has abdominal pain, mucus, and blood in stool, and grade 2 diarrhea is four to six stools per day over baseline. Grade 3 colitis has severe pain, fever, and peritoneal signs, and grade 3 diarrhea is ≥7 stools per day. Grade 4 colitis has life-threatening consequences such as perforation, ischemia, necrosis, bleeding, and toxic megacolon. Grade 4 diarrhea also has life-threatening consequences such as hemodynamic collapse [6]. Grade 5 colitis and diarrhea signify the death of the patient secondary to these irAEs. For the current case, this patient most likely had grade 2 colitis and grade 3 diarrhea at presentation due to abdominal pain, mucus, and blood in the stool as well as >7 stools per day without any overt life-threatening consequences such as perforation, ischemia, necrosis, and hemodynamic instability.

Treatment for colitis and diarrhea secondary to ICIs depends on the severity of the disease as elucidated by the grade of the disease. Different societies, such as the Society for Immunotherapy of Cancer, the American Society of Clinical Oncology, and the European Society for Medical Oncology, have different treatment plans depending on the grade of colitis and diarrhea. The consensus is that first-line treatment is systemic steroids, followed by TNF-alpha inhibitors such as infliximab if symptoms do not improve [6]. In more severe cases of ICI-induced colitis and diarrhea, a multidisciplinary approach involving a team of specialists may be necessary. These specialists may include oncologists, gastroenterologists, and immunologists, who will collaborate to develop a personalized treatment plan for the patient.

This case illustrates the debilitating adverse effects of pembrolizumab and how it can mimic IBD and enteric infections. The general workup for ICI-induced colitis should rule out an infectious cause with culture as well as stool ova and parasite. Stool inflammatory markers such as stool WBC and fecal calprotectin can also be assessed with a stool culture. Colonoscopy following inflammatory bowel markers can provide a definitive diagnosis as to the extent of colitis and can help rule out mimics such as IBD. In the current case, infectious etiologies were ruled out early on; however, significantly elevated inflammatory markers and pancolitis on colonoscopy indicated an inflammatory etiology to the patient's presentation. Furthermore, the decrease in bowel movements after discontinuing the ICIs pointed toward ICI-induced colitis. Treatment for this patient was initiated with a trial of IV steroids for five days, followed by oral steroids with an outpatient taper once the patient’s bowel movements were controlled to <4 per day.

## Conclusions

Pembrolizumab-induced colitis and diarrhea are known but rare adverse effects of immunotherapy. The current case demonstrates the severity of immunotherapy-induced colitis. However, for patients on any immunotherapy presenting with signs and symptoms of severe diarrhea, an infectious etiology must be ruled out in addition to working up an inflammatory cause of the diarrhea. Treatment for ICI-induced colitis and diarrhea includes discontinuing the offending agent, followed by steroid treatment and/or TNF-alpha inhibitors. Different academic societies have varying guidelines on treatment plan escalation based on the grade of colitis and diarrhea. Understanding immunotherapy-related adverse events like colitis and diarrhea remains an evolving field, and continued research will allow for more effective treatment strategies for patients experiencing these challenges.
